# Pulmonary Tuberculosis

**DOI:** 10.1155/2013/645747

**Published:** 2013-04-14

**Authors:** Anete Trajman, José R. Lapa e Silva, Margareth Dalcolmo, Jonathan E. Golub

**Affiliations:** ^1^Gama Filho University, Rua Manoel Vitorino 553, Prédio AG, 5° andar, 20740-900 Rio de Janeiro, RJ, Brazil; ^2^Chest Institute, McGill University, 3650 St. Urbain Street, Montreal, PQ, Canada H2X 2P4; ^3^Federal University of Rio de Janeiro, Avenida Professor Rodolpho Paulo Rocco 255, 21941-913 Rio de Janeiro, RJ, Brazil; ^4^Center for Global Health, Weill Medical College of Cornell University, New York, NY, USA; ^5^Centro de Referência Professor Helio Fraga, FIOCRUZ, Estrada de Curicica 2000, CEP 22780-192 Rio de Janeiro, RJ, Brazil; ^6^Johns Hopkins University School of Medicine and Bloomberg School of Public Health, Center for TB Research, 1550 Orleans Street, CRB-2, Room 1M.10, Baltimore, MD 21231, USA

Tuberculosis (TB) is a major killer worldwide. The World Health Organization estimates that in 2011, there were 8.7 million incident cases of TB, 1.4 million deaths from TB including 0.43 million deaths from HIV-associated TB. In recent years, after a century of stagnation, new diagnostic technologies have been developed, and they are already being scaled up in some high-burden countries. Likewise, new drugs and regimens to treat TB are being evaluated, and the development of new vaccines is also progressing. However, despite the hope for reduced transmission with earlier detection and effective treatment, new cases continue to emerge from latently infected individuals.

In the present issue, P. Narasimhan et al. review risk factors for progression to active disease. Immunological aspects of TB are discussed in three papers, with J.-G. Ocejo-Vinyals et al. indicating that mannose-binding lectin 2 promoter polymorphisms and gene variants are not associated with an increased risk of pulmonary tuberculosis in a genetically conserved population in Spain; Y. V. N. Cavalcanti et al. reviewed the role of cytokines in protective immunity and susceptibility to tuberculosis; I. Takenami et al. provide evidence that blood IFN-gamma levels in tuberculin skin test positive individuals increase after six months of isoniazid treatment.

In addition, several papers evaluate the new interferon-gamma release assays (IGRAs), which have replaced or complemented the tuberculin skin test in many developed countries in the last decade. A. Trajman et al. review the current clinical uses of these tests. S. S. Shin et al. used IGRAs to investigate the effect of cigarette smoking on TB transmission, and W. Thanassi et al. explore the utility of IGRAs for serial testing in healthcare workers.

We believe that this issue can contribute to the debate of relevant topics concerning new advances on tuberculosis among pulmonologists, infectologists, clinicians, epidemiologists, and basic science researchers.

*Anete Trajman* *Anete Trajman* *José R. Lapa e Silva* *José R. Lapa e Silva* *Margareth Dalcolmo* *Margareth Dalcolmo* *Jonathan E. Golub* *Jonathan E. Golub*

